# Antifibrotic therapy by sustained release of low molecular weight heparin from poly(lactic-co-glycolic acid) microparticles on bleomycin-induced pulmonary fibrosis in mice

**DOI:** 10.1038/s41598-020-76034-0

**Published:** 2020-11-04

**Authors:** Takashi Saito, Takuya Kotani, Koichi Suzuki

**Affiliations:** 1 Department of Legal Medicine, Osaka Medical College, 2-7 Daigakumachi, Takatsuki, Osaka 568-8686 Japan; 2 Division of Rheumatology, Department of Internal Medicine IV, Osaka Medical College, Takatsuki, Osaka Japan

**Keywords:** Cell biology, Drug delivery

## Abstract

Heparin and low molecular weight heparin (LMWH) have recently been considered useful treatment tools for inflammation. Heparin has antifibrotic activity, mediated by cellular secretion of hepatocyte growth factor (HGF). HGF has antifibrotic properties demonstrated in experimental models of lung, kidney, heart, skin, and liver fibrosis. The ability of LMWH for HGF secretion is similar to that of normal heparin. Poly (lactic-co-glycolic acid) (PLGA) is widely used for sustained drug release, because of its biocompatibility and low toxicity. LMWH-loaded PLGA microparticles are prepared by a conventional water-in-oil-in-water emulsion method. Interstitial pneumonia is a life-threatening pathological condition that causes respiratory failure when it progresses. In the present study, we investigated the therapeutic effect of LMWH-loaded PLGA microparticles in a mouse model of bleomycin-induced lung fibrosis. The ratios of fibrotic area to total area were significantly lower in mice administered LMWH-loaded microparticles than in mice administered bleomycin alone. The microparticle administration did not further enhance the gene expression for inflammatory cytokines. In a cell culture study, HGF secretion by mouse and human lung fibroblasts was significantly increased by LMWH addition. We conclude that LMWH showed anti-inflammatory activity, through the effects of LMWH-loaded PLGA microparticles on cells at sites of inflammation.

## Introduction

Interstitial pneumonia (IP) is a life-threatening pathological condition that causes respiratory failure when it progresses. Fibrosis is the excess production of collagens and other proteins as a result of long term inflammation^1^. There are several types of IP with varying characteristics: fibrosis is the main pathology in usual IP represented by idiopathic pulmonary fibrosis (IPF), both interstitial inflammation and fibrosis develop in the lungs in non-specific IP commonly found in connective tissue disease, and fibrosis is induced by rapid pulmonary interstitial inflammation in diffuse alveolar damage^2^. Lung inflammation is treated with corticosteroids and immunosuppressants like cyclosporine, cyclophosphamide, azathioprine, and mycophenolate mofetil^3,4^, while pulmonary fibrosis is treated with antifibrotic agents like pirfenidone and nintedanib^5^. In IPF, pirfenidone and nintedanib prolong survival by delaying the progression of lung fibrosis, but their effects remain limited. Many patients are treatment-resistant and the outcomes are poor in all disease types, which is problematic. New therapeutic developments for lung fibrosis are required.

Heparin is a glycosaminoglycan composed of alternating glucosamine and glucuronic residues. It has been clinically used as an anticoagulant agent, but has the side effect of bleeding acceleration^6^. Compared with normal heparin, low molecular weight heparin (LMWH) can induce less bleeding^7^. Heparin was also shown to have antifibrotic activity mediated by cellular secretion of hepatocyte growth factor (HGF)^8,9^. The ability of LMWH for HGF secretion is similar to that of normal heparin^10^. Furthermore, controlled release of LMWH achieved therapeutic efficacy against peritoneal fibrosis^11^. Based on these backgrounds, a controlled release system was chosen for evaluation of the antifibrotic effects of fragmin (dalteparin sodium aqueous solution), a type of LMWH, in the present study.

Poly (lactic-co-glycolic acid) (PLGA) nanoparticles and microparticles are widely used for sustained drug release^12,13^, because of their biocompatibility and low toxicity^14^. PLGA is biologically degraded by the surrounding cells through normal metabolic pathways^15^. The degradation profile can be changed by the ratio of lactic acid and glycolic acid^16,17^. Indeed, several therapies using PLGA and combinations with other carriers encapsulating bioactive molecules have recently entered preclinical development^18,19^.

In the present study, LMWH-loaded PLGA microparticles (LMWH/PLGA-MPs) were prepared for controlled release of LMWH. PLGA-5005 (number average molecular weight: 5,000; L:G = 50:50) was chosen for the evaluation. We examined the therapeutic effect of intravenous LMWH/PLGA-MPs on mice with bleomycin (BLM)-induced IP (BLM-IP), and further investigated the possible mechanism for the favorable effect of LMWH on BLM-IP by focusing on HGF because of its antifibrotic effect in IP.

## Materials and methods

### Ethics

The Institutional Animal Care and Use Committee of Osaka Medical College approved all of the research protocols (approval ID: 2019-11), including the surgical procedures and animal care, and all methods were performed in accordance with the relevant guidelines and regulations.

### Materials

Collagen type I aqueous solution was kindly supplied by Nitta Gelatin Co. (Osaka, Japan). Human pulmonary fibroblasts (HPFs), fibroblast growth medium 2 (FGM2), and fetal bovine serum (FBS) were purchased from Takara Bio Inc. (Shiga, Japan). Cell Count Reagent SF and HEPES were purchased from Nacalai Tesque Inc. (Kyoto, Japan ). A mouse lung fibroblast cell line (Mlg2908), a human alveolar adenocarcinoma cell line (A549), and a mouse alveolar epithelial cell line (MLE-12) were purchased from ATCC (VA, USA). F12K media were purchased from ATCC (VA, USA). L-Glutamine, solution (200 mM) was purchased from (Thermo Fisher Scientific Inc., MA, USA). Insulin, sodium selenite and β-estradiol were purchased from Sigma-Aldrich Japan K.K. (Tokyo, Japan). A Transferrin was purchased from BD Japan (Tokyo, Japan). A hydrocortisone was purchased from Tokyo Chemical Industry Co., Ltd. (Tokyo, Japan). A mixture of 10,000 U penicillin and 10 mg/mL streptomycin (Pen-Strep) and poly(vinyl alcohol) (PVA; average molecular weight: 85,000–124,000; 87%–89% hydrolysed) were purchased from Merck KGaA (Darmstadt, Germany). Dichloromethane (DCM), DMEM/Ham’s F-12 (DMEM/F12), PLGA-5005, and 4% paraformaldehyde in phosphate-buffered saline (4% PFA/PBS) were purchased from Fujifilm Wako Pure Chemical Corporation (Osaka, Japan). Fragmin was purchased from Kissei Pharmaceutical Co. Ltd. (Tokyo, Japan).

### Cell culture

HPF and Mlg2908 cells were cultured in FGM2 and DMEM/F-12 containing 10% FBS and 1.0% Pen-Strep, respectively. A549 cells were cultured in F12K medium containing 10% FBS. MLE-12 cells were cultured in DMEM/Ham’s F-12 containing 2% FBS, L-glutamine (2 mM), insulin (5 ug/mL), transferrin (10 µg/mL), sodium selenite (30 nM), Hydrocortisone (10 nM), β-estradiol (10 nM), HEPES (10 mM). The wells of 96-well multiwell culture plates (Sumitomo Bakelite Co. Ltd., Tokyo, Japan) were coated with collagen type I aqueous solution. Briefly, 1% collagen solution (pH 3.0) was placed in each well and incubated for 10 min. After removal of the solution, the wells were dried for 30 min and washed twice with PBS (pH 7.4; 100 µL). Cells were seeded into the coated wells at 5000 cells /well in medium 100 (µL) supplemented with or without LMWH, and cultured for 24 h at 37 °C in an atmosphere of 5% CO_2_/95% air. The cell numbers were counted using Cell Count Reagent SF according to the manufacturer’s protocol. Briefly, the medium was exchanged for 100 µL of fresh medium containing 2-(2-methoxy-4-nitrophenyl)-3-(4-nitrophenyl)-5-(2,4-disulfophenyl)-2H-tetrazolium and further incubated for 1 h. The absorbance of the medium was measured at 450 nm. The percentage of cell viability was expressed relative to 100% for cells cultured without fragmin.

### HGF immunoassay

Supernatants were evaluated for their HGF contents using an HGF ELISA Quantikine Kit (R&D Systems Inc., Minneapolis, MN, USA) according to the manufacturer’s protocol. The experiments were carried out six times independently for each sample, and in duplicate.

### Microparticle preparation

Fragmin solution (1000 IU/mL; 200 µL) was mixed with DCM containing PLGA (50 mg/2 mL) and 2% PVA (2 mL) for 60 s at 3000 rpm using a vortex to create the first emulsion (W_1_/O). The first emulsion was poured into a 2% PVA aqueous solution (16 mL) and mixed with a vortex mixer for 60 s. This procedure permitted the formation of a double emulsion (water-in-oil-in-water: W_1_/O/W_2_), in which the W_1_ phase was homogeneously dispersed in the O phase. PVA aqueous solution (20 mL) was then added, and the W_1_/O/W_2_ emulsion was continuously stirred for 6 h at room temperature until the DCM had completely evaporated. The microparticles were filtered through a 40-µm cell strainer (Corning, New York, NY, USA) and washed with double-distilled water by centrifugation three times. The mixture was freeze-dried in a freeze drier (VD-550R; Taitec, Saitama, Japan) to produce powdered microparticles.

### Scanning electron microscopy observation

LMWH/PLGA-MPs were fixed on an aluminum support with carbon-adhesive glue and coated with a 10-nm thick layer of palladium using an ion sputter (30 mA; E-1030; Hitachi Ltd., Tokyo, Japan). The samples were observed using a scanning electron microscope (S-5000; Hitachi Ltd.). LMWH/PLGA-MPs size was measured by ImageJ (n = 1,617).

### Surgical procedure

Female 13–14-week-old ICR mice (Shimizu Laboratory Supplies, Kyoto, Japan) were anesthetised with an intraperitoneal injection of 400 mg/kg 2,2,2-tribromoethanol (Avertin; Sigma-Aldrich Japan K.K., Tokyo, Japan), and divided into three groups : mice with no treatment (Normal); mice with BLM-IP (BLM-alone); and mice with LMWH/PLGA-MP treatment (BLM-LMWH). All experiments used five mice/group. The administered BLM solution was prepared by mixing sterile BLM sulfate powder (Nippon Kayaku, Tokyo, Japan) with sterile normal saline. A dose of 5 mg BLM in a total volume of 100 µL of sterile saline was injected subcutaneously using an osmotic minipump (Alzet 2010; DURECT, Cupertino, CA, USA) from day 0 to day 7. After the BLM administration, LMWH/PLGA-MPs in PBS (100 µg/mL) were injected three times via a tail vein on days 7, 14, and 21. Mice receiving the same volume of PBS without LMWH/PLGA-MPs were used as controls. The mice were euthanised on day 21 (total: 28 days) after LMWH/PLGA-MP/PBS injection, and their lungs were harvested for histological analysis.

### Histological analysis and assessment of fibrotic area

The right middle lung of each mouse was fixed for 6 h in 4% PFA/PBS followed by overnight incubation in 20% sucrose/PBS. The tissues were embedded in OCT compound (Sakura FineTek, Tokyo, Japan), cut into 5-µm sections, and processed for hematoxylin and eosin (H&E) or Masson’s trichrome staining for analysis of morphological or fibrotic changes. For each section, the five most severely injured non-overlapping fields (magnification: 200×) of the lung parenchyma were evaluated. Quantification of lung fibrosis in the histological specimens was performed using a numerical scale (modified Ashcroft score)^20^. The severity of the fibrotic changes in each microscopic field of a given lung section was assessed and assigned a modified Ashcroft score from 0 to 8. The overall severity in each lung section was expressed as the mean score for the examined microscopic fields. All histological examinations were assessed by three independent observers. The blue area after Masson’s trichrome staining was determined as the fibrotic area. The fibrotic area/total area ratio was measured by a WinROOF Ver 6.1 computerised morphometry system (Mitani, Fukui, Japan) for each section.

### Quantitative real-time RT-PCR (qPCR)

Total RNA was extracted from lungs of mice at 21 days after receiving BLM (n = 5 per group). After RNA extraction with an RNeasy Mini Kit (Qiagen Ltd., Manchester, UK), cDNA was synthesised using an ExScript RT Kit (Takara, Shiga, Japan) and amplification was performed in a Sequence Detection System 7000 (Thermo Fisher Scientific Inc., MA, USA) according to the manufacturer’s instructions. The primer sequences for *matrix metalloproteinase-2* (*MMP-2*), *matrix metalloproteinase-9* (*MMP-9*), *tissue inhibitor of metalloproteinase-1* (*TIMP-1*), *tissue inhibitor of metalloproteinase-2* (*TIMP-2*), *collagen type 1 alpha 1* (*COL1A1*), *hepatocyte growth factor* (*HGF*), *interleukin-1 beta* (*IL-1β*), *interleukin-6* (*IL-6*), *interleukin-12* (*IL-12*), *tumor necrosis factor-α* (*TNF-α*), and *glyceraldehyde 6-phosphate dehydrogenase (GAPDH)* as a housekeeping gene are summarised in Table 1. The amount of each target gene expression was normalised to the housekeeping gene expression. The following PCR conditions were used: 95 °C for 3 min, followed by 40 cycles of 95 °C for 10 s, 56 °C for 30 s, and 95 °C for 10 s, and a final extension at 95 °C for 50 s. The relative mRNA expression level of each target gene was calculated by the comparative CT method^21^. The experiments were carried out five times independently for each sample, and in duplicate.

**Table 1:** Primers used in the qPCR analyses.

Gene	Forward	Reverse
*MMP-2*	5′-AACCTCTTTGTGCTGAAA-3'	5′-GATGGTGTTCTGGTCAAG-3'
*MMP-9*	5′-CGATTCCAAACCTTCAAA-3'	5′-GCAAGTCTTCAGAGTAGT-3'
*TIMP-1*	5′-AAGATGACTAAGATGCTAA-3'	5′-GATGAGAAACTCTTCACT-3'
*TIMP-2*	5′-CTCGGAGCGCAATAAAACGG-3'	5′-CCTCTTGATGGGGTTGCCAT-3'
*COL1A1*	5′-AAGAAGACATCCCTGAAG-3'	5′-ATACAGATCAAGCATACCT-3'
*HGF*	5′-CGCGGATGGTTTATTACGA-3'	5′-TCTTTTCAGCCCCAGCAC-3'
*IL-1β*	5′-GATACCACTCCCAACAGA-3'	5′-GCCATTGCACAACTCTTT-3'
*IL-6*	5′-GATACCACTCCCAACAGA-3'	5′-GCCATTGCACAACTCTTT-3'
*IL-12*	5′-AAGATGAAGGAGACAGAG-3'	5′-CATTGGACTTCGGTAGAT-3'
*TNF-α*	5′-TCTCCCTGATCGGTGACAGT-3'	5′-GGGCAGAGCTGAGTGTTAGC-3'
*GAPDH*	5′-ACAATGAATACGGCTACAG-3'	5′-GGTCCAGGGTTTCTTACT-3'

### Statistical analysis

All results are expressed as mean ± standard deviation (SD). Data were analysed by non-parametric one-way ANOVA followed by a multiple-comparisons test. Differences were considered significant for values of *p* < 0.05. All statistical analyses were performed using GraphPad Prism 7.04 software (GraphPad Software, San Diego, CA, USA).

## Results

### Cell viability

**Figure 1:**
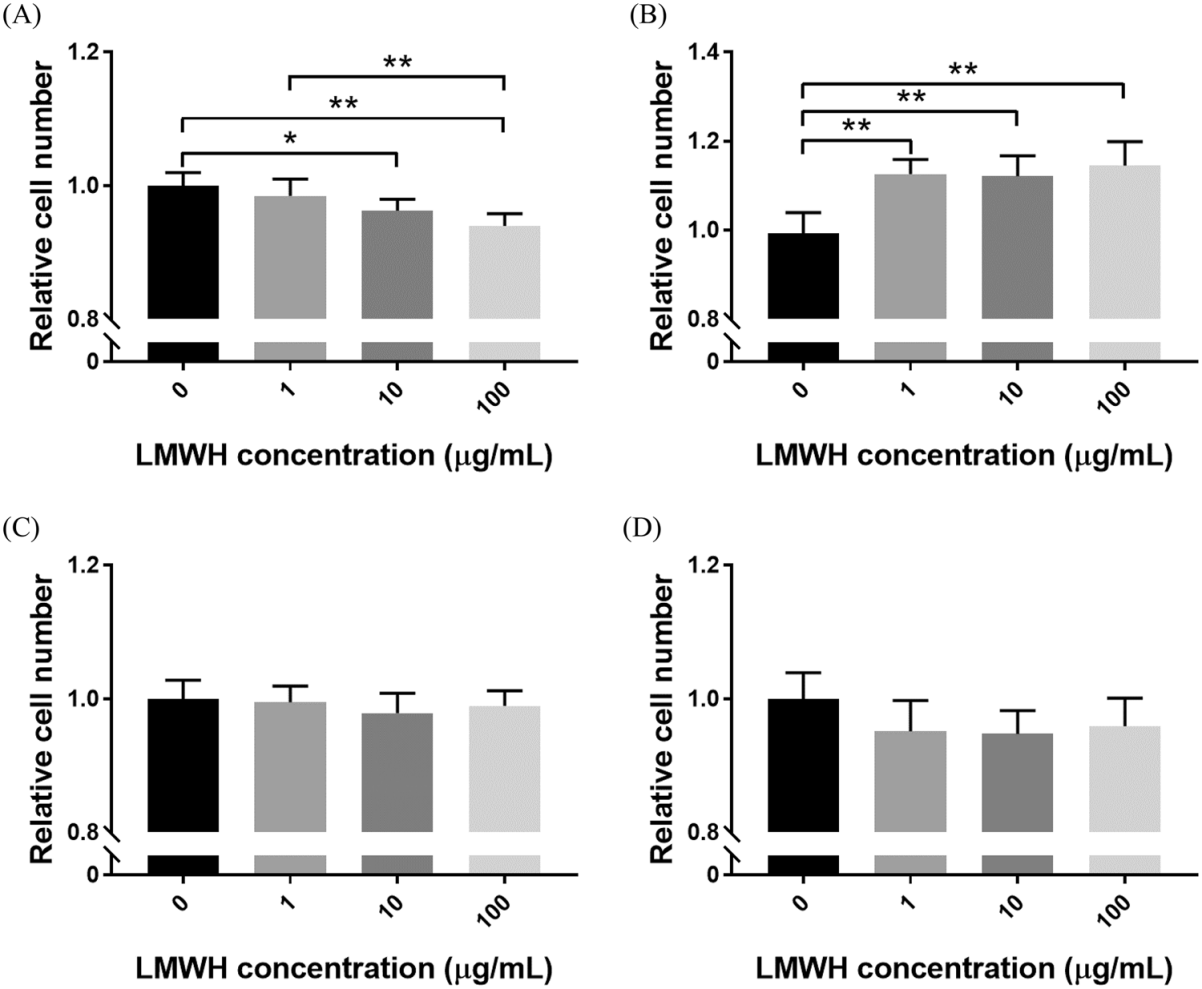
Cell viabilities in the presence of LMWH. Mlg2908 (**A**), HPF (**B**), MLE-12 (**C**) and A549 (**D**) cells were cultured in medium supplemented with or without LMWH, and measured for their cell viabilities. *p < 0.05, **p < 0.01, significant difference between the linked groups.

Figure 1 shows the viabilities of Mlg2908 (A), HPF (B), MLE-12 (C) and A549 (D) cells in the presence of LMWH. As shown in Fig. 1A, LMWH showed no cytotoxicity toward Mlg2908 cells at a low concentration (1 µg/mL), but showed significant cytotoxicity toward Mlg2908 at higher concentrations (10 and 100 µg/mL). HPF viability was significantly higher in the presence of LMWH compared with the absence of LMWH (Fig. 1B). As shown in Fig. 1C,D, LMWH showed no cytotoxicity toward MLE-12 and A549 cells.

### HGF secretion

**Figure 2:**
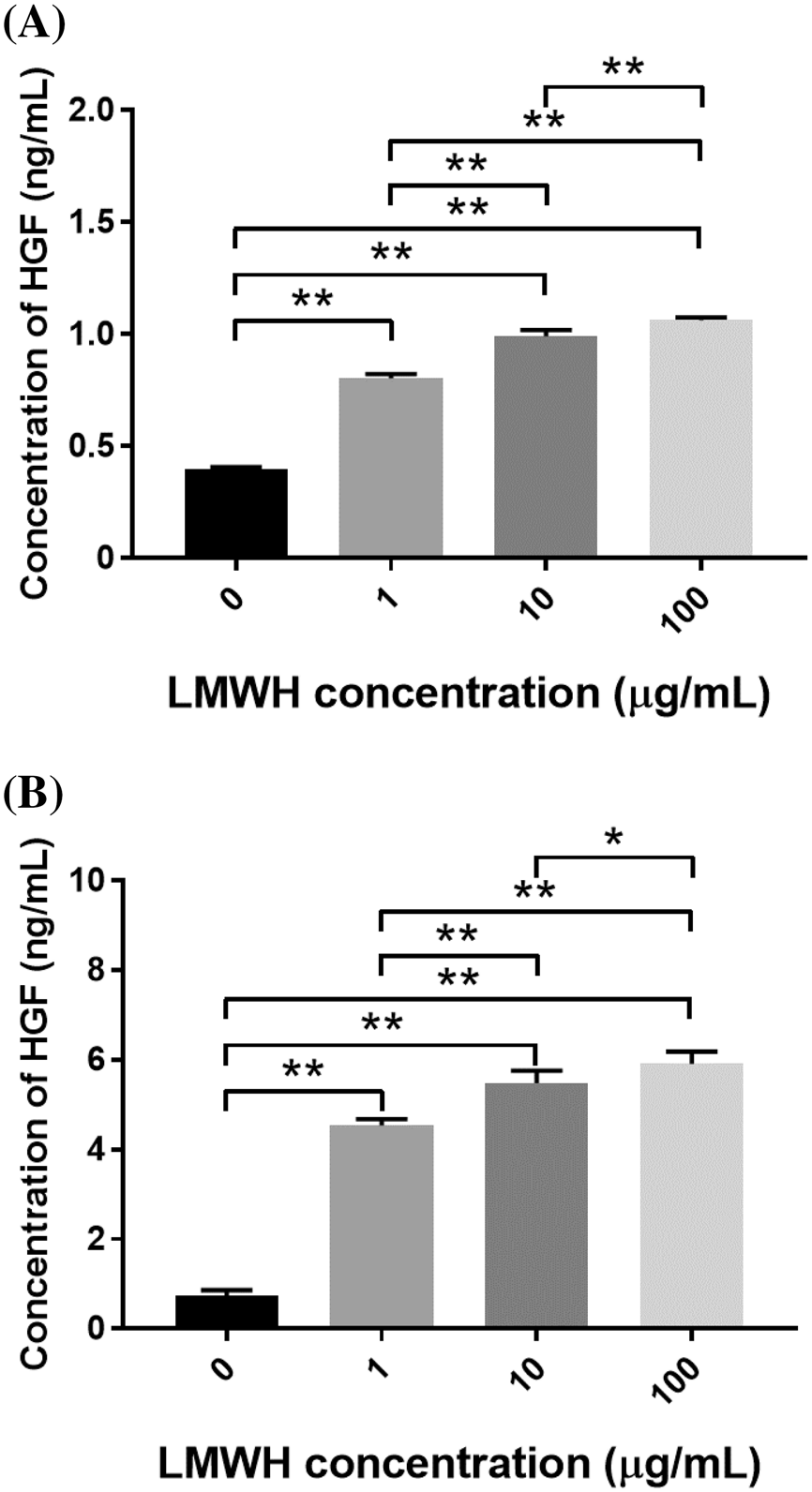
Human and mouse HGF secretion is up-regulated in the presence of LMWH. (A, B) The amounts of HGF secreted by Mlg2908 (**A**) and HPF (**B**) cells cultured with different concentrations of LMWH were measured. *p < 0.05, **p < 0.01, significant difference between the linked groups.

Figure 2 shows the amounts of HGF secretion. The amounts of HGF secreted by Mlg2908 (Fig. 2A) and HPF (Fig. 2B) cells were significantly increased after LMWH addition.

### Microparticles observation

**Figure 3:**
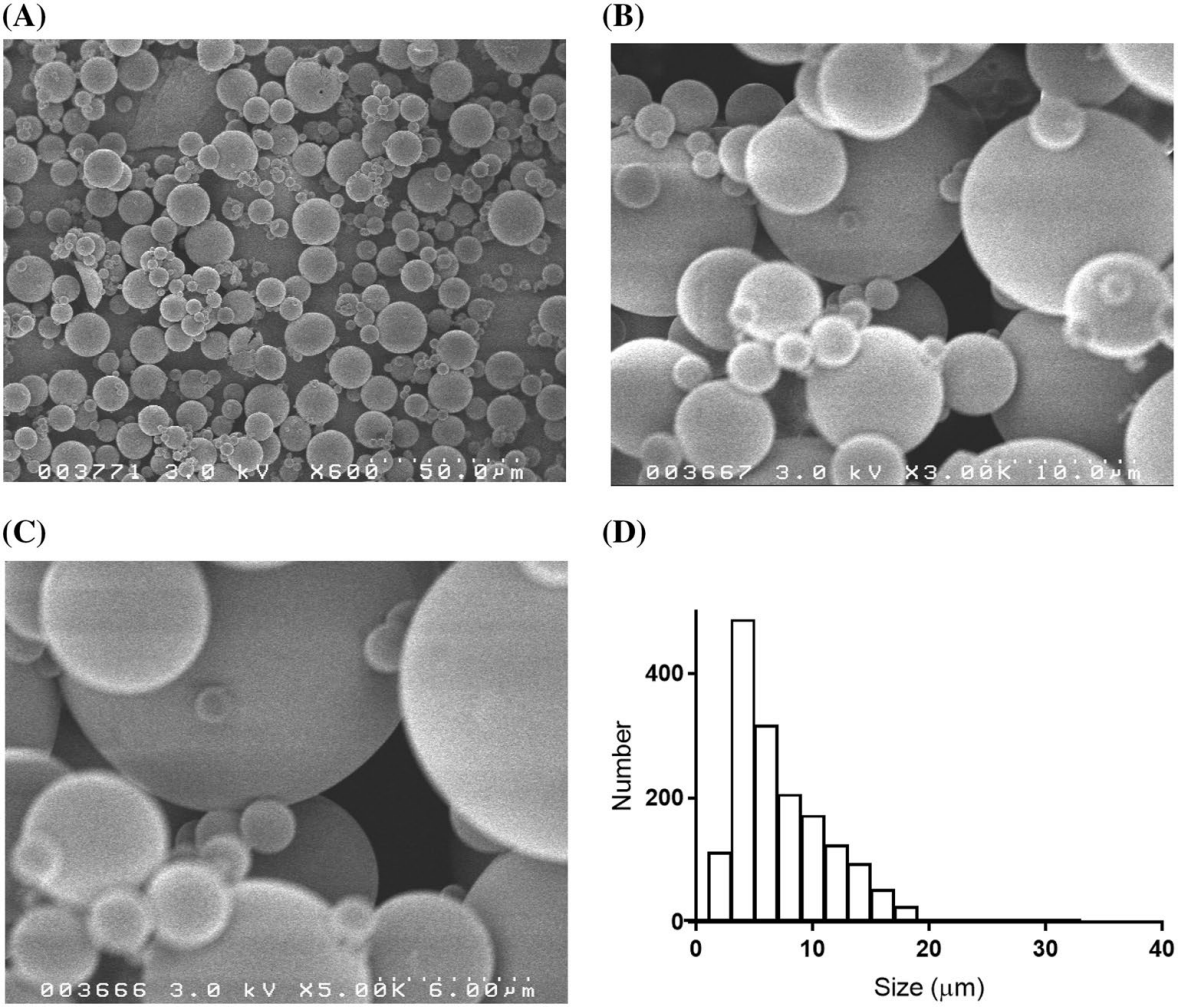
SEM of LMWH/PLGA-MPs. (**A**–**C**) SEM images of LMWH/PLGA-MPs at different magnifications are shown. (**D**) LMWH/PLGA MPs size distribution is shown.

Figure 3A–C shows images of LMWH/PLGA-MPs at different magnifications. The microparticle size distribution were shown in Fig. 3D. The microparticles size were 6.4 ± 4.2 µm. The minimam and maximan size of the microparticles were 0.97 and 31.2 µm, respectively. The observations confirmed that microparticles of less than 40 µm were prepared.

### LMWH/PLGA-MPs reduce lung fibrosis

**Figure 4:**
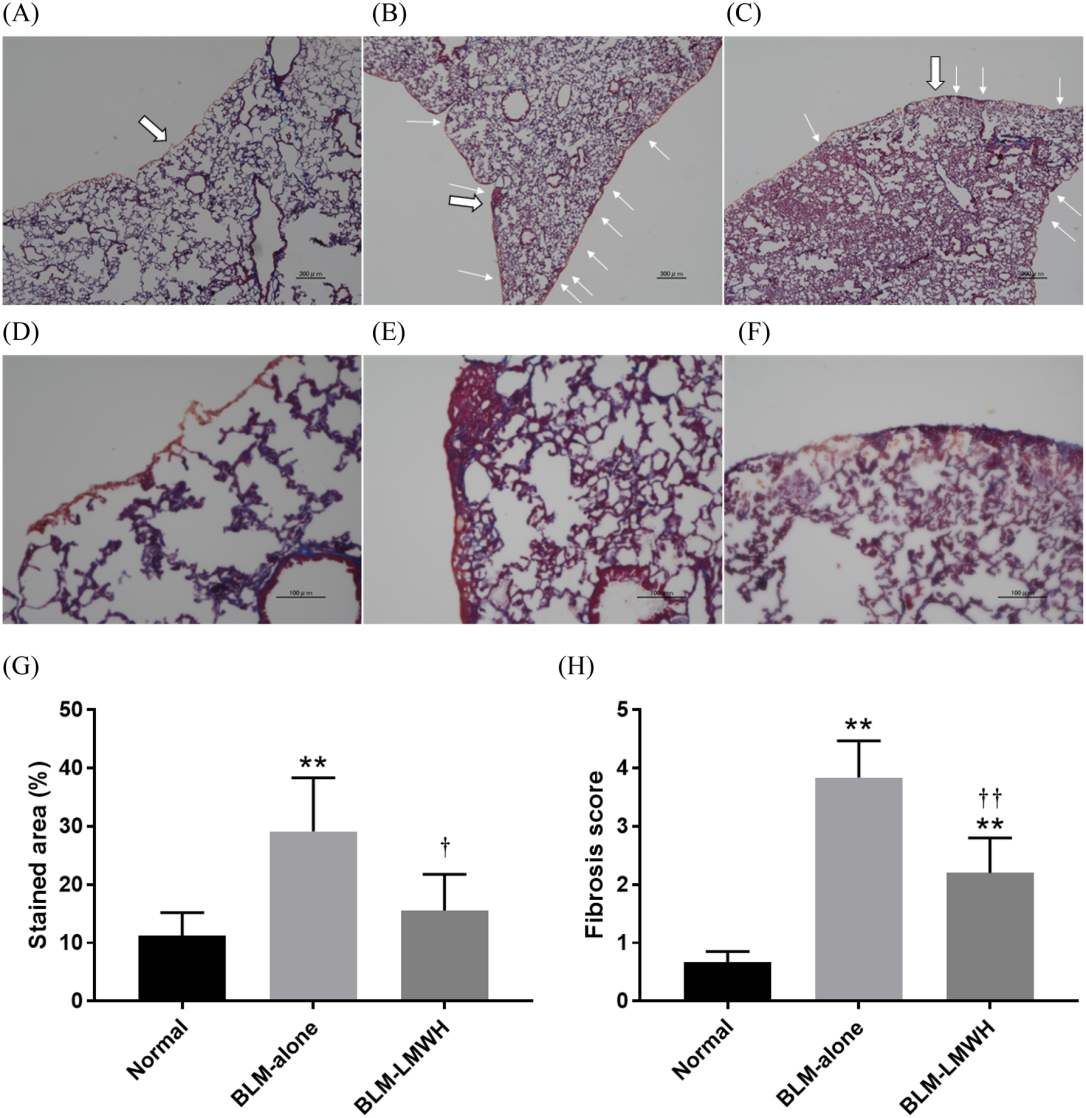
Administration of LMWH/PLGA-MPs reduces lung fibrosis. (**A**–**F**) Histological sections of lung tissues from mice in the normal (**A**, **D**), BLM-alone (**B**, **E**), and BLM-LMWH (**C**, **F**) groups at 28 days after application with or without LMWH/PLGA-MPs. The Figure (**A**–**C**) and (**D**–**F**) are ×40 and ×200 magnification, respectively. The Figures (**D**–**F**) are enlarged images of the large write arrow parts in the Figures (**A**–**C**). Scale bars: 300 µm (**A**–**C**) and 100 µm (**D**–**F**). Collagen deposition areas (**G**) and fibrotic areas (**H**) at 28 days after application with or without LMWH/PLGA-MPs. *p < 0.05, **p < 0.01, significant difference versus the normal group. ^†^*p* < 0.05, ^††^*p* < 0.01, significant difference versus the BLM-alone group.

Representative lung sections were subjected to Masson’s trichrome staining at 28 days after starting BLM administration (Fig. 4A–F). The continuous subcutaneous infusion of BLM using an osmotic minipump forms fibrotic lesions with collagen deposition mainly on pleural side (small write arrows). The lung sections in the BLM-alone group showed a diffuse increase in collagen deposition (Fig. 4B,E) compared with the normal lung sections (Fig. 4A,D). The increase in collagen deposition in the lung was reduced by administration of LMWH/PLGA-MPs (Fig. 4C,F). The fibrotic area/total area ratio (Fig. 4G) and fibrosis score (Fig. 4H) were significantly higher in the BLM-alone group than in the normal group, but significantly lower in the BLM-LMWH group compared with the BLM-alone group.

### LMWH/PLGA-MPs reduce fibrosis in the BLM-IP lung

**Figure 5:**
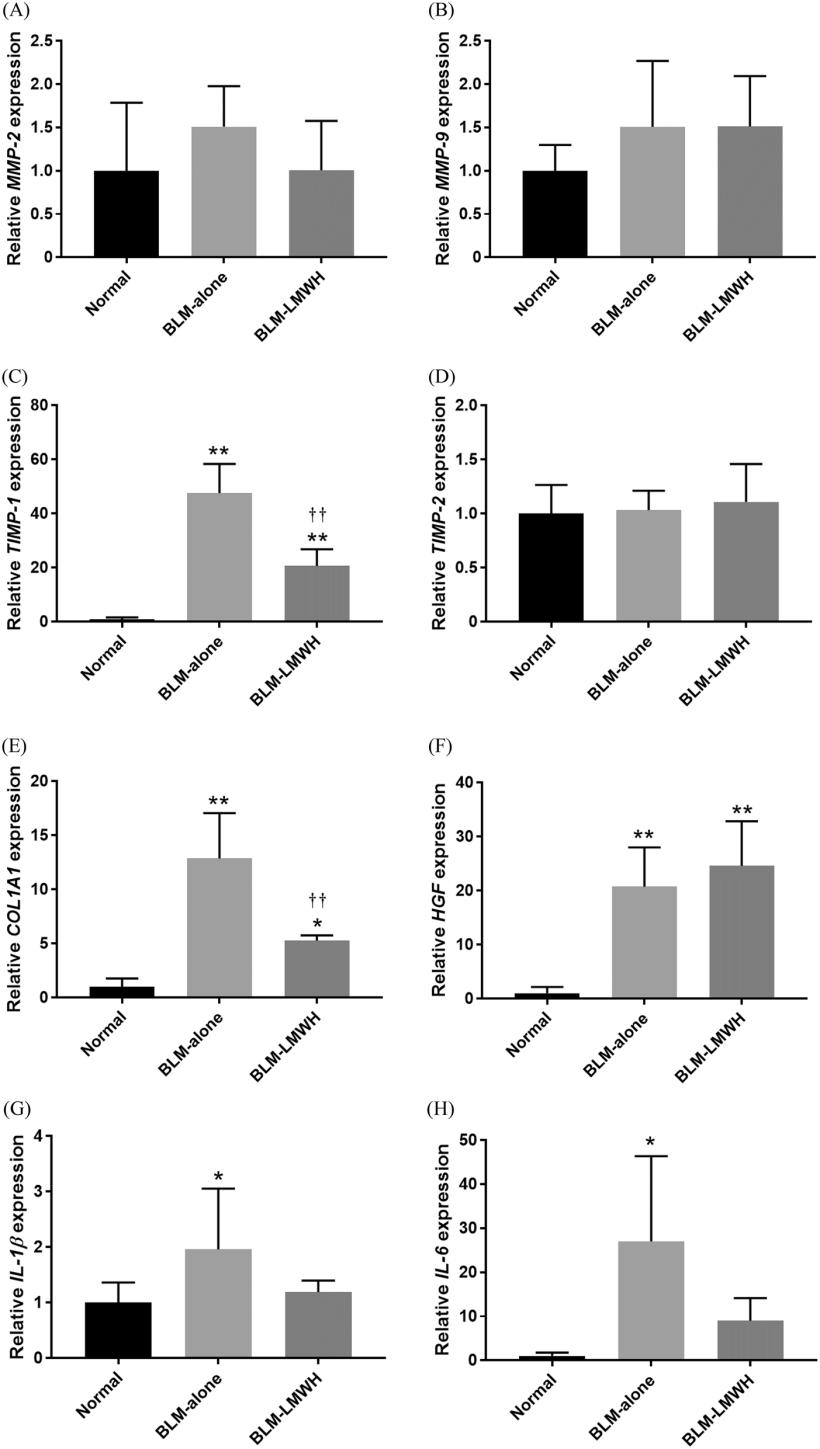

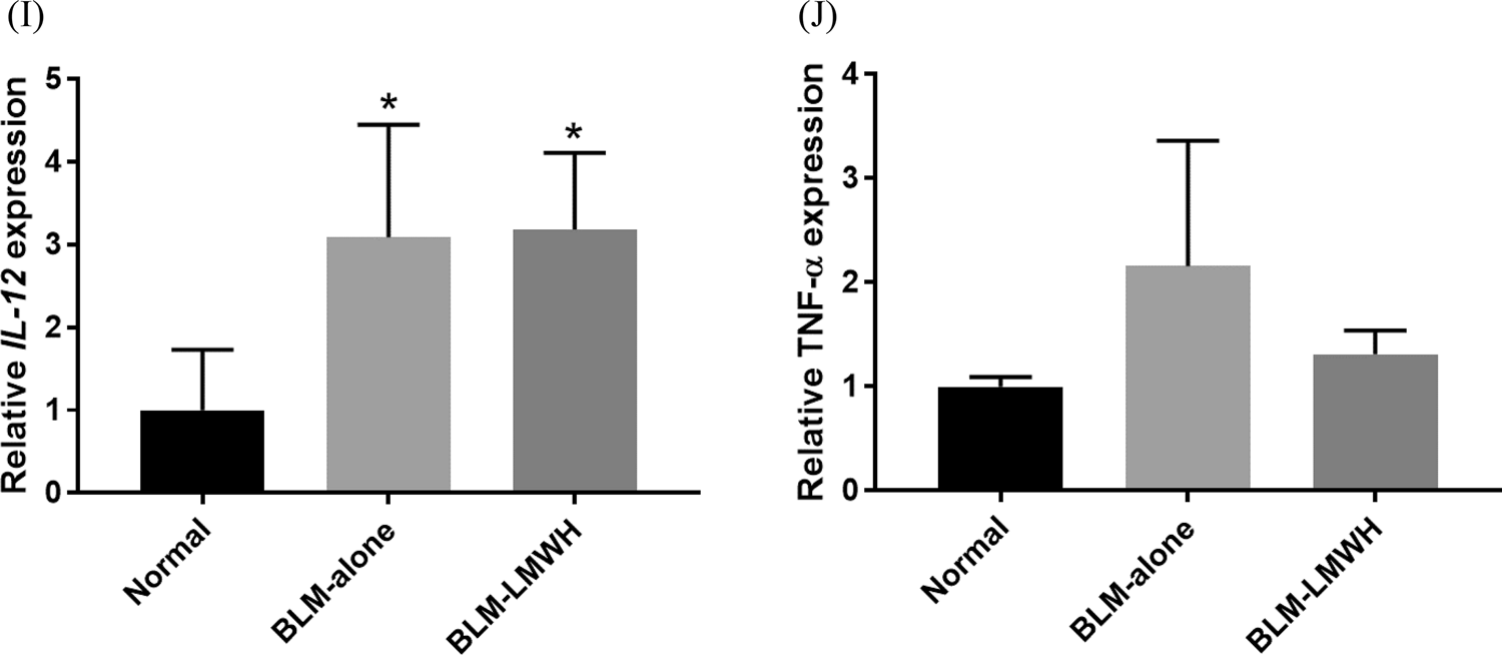
Quantitative real-time RT-PCR analysis of whole-lung mRNA expression. (**A**–**J**) Relative mRNA expression levels of *MMP-2* (**A**), *MMP-9* (**B**), *TIMP-1* (**C**), *TIMP-2* (**D**), *COL1A1* (**E**), *HGF* (**F**), *IL-1β* (**G**), *IL-6* (**H**), *IL-12* (**I**), and *TNF-α* (**J**) in the lungs of mice at 28 days after administration of BLM. *p < 0.05, **p < 0.01, significant difference versus the normal group. ^†^*p* < 0.05, ^††^*p* < 0.01, significant difference versus the BLM-alone group.

To determine whether the decrease in collagen was caused by reduced synthesis, whole lung mRNA expression was analysed by quantitative real-time PCR for *COL1A1*, *TIMP-1*, *TIMP-2*, *MMP-2*, *MMP-9*, and *HGF* (Fig. 5A–F). Significant increases in *COL1A1* and *TIMP-1* relative mRNA expression levels were observed at 28 days after BLM administration. The *COL1A1* and *TIMP-1* relative mRNA expression levels at 28 days after starting BLM administration were significantly lower in the BLM-LMWH group compared with the BLM-alone group. There were no significant changes in the *TIMP-2*, *MMP-2*, *MMP-9*, and *HGF* relative mRNA expression levels among the groups.

### Evaluation of anti-inflammatory effects

To evaluate the anti-inflammatory effects of LMWH, whole lung mRNA expression was analysed by quantitative real-time RT-PCR for the following inflammatory cytokines: *IL-1β, IL-6*, *IL-12*, and *TNF-α* (Fig. 5G–J). There were significant increases in the *IL-1β*, *IL-6*, and *IL-12* relative mRNA expression levels and a tendency toward an increase in the *TNF-α* relative mRNA expression levels at 28 days after BLM administration. However, there were no significant changes in the *IL-1β*, *IL-6*, *IL-12*, and *TNF-α* relative mRNA expression levels in the BLM-LMWH group compared with the BLM-alone group.

## Discussion

This study demonstrates that LMWH showed anti-inflammatory activity, through the effects of LMWH/PLGA-MPs preventive against fibrogenesis in the BLM-IP mice. The Mlg2908 and HPF cells had served as models of pulmonary fibroblasts. The MLE-12 and A549 cells had served as models of alveolar Type II pulmonary epithelium. The LMWH showed significant cytotoxicity toward Mlg2908 cells at higher concentrations (10 and 100 µg/mL). However, the amounts of HGF secretion were significantly increased by LMWH addition (Fig. 2A). It may be considered that the LMWH simply suppressed proliferation of Mlg2908 cells and increased HGF secretion at higher concentrations (10 and 100 µg/mL). There were no significant differences in the *HGF* relative mRNA expression levels between the BLM-alone and BLM-LMWH groups. However, in the in vitro cell culture experiments, the amounts of HGF secretion were significantly increased by LMWH addition. Similar to our results, there are several reports that heparin or LMWH increased HGF secretion without affecting *HGF* relative mRNA expression levels^9,22^. These results suggest that LMWH may affect the promoter region of the *HGF* gene without increasing the gene expression^10^. Activation of HGF resulted in phosphorylation of the HGF receptor (c-Met)^23^. Furthermore, the anti-fibrotic effect of the HGF/Met pathway is not restricted to the lung and has been consistently demonstrated in experimental models of liver, kidney, heart, and skin fibrosis^24–29^. The roles of HGF/met signaling pathway have been reported. Administration of HGF to bleomycin-induced pulmonary fibrosis in mice increased lung MMP activities and enhanced myofibroblast apoptosis^30^. HGF also induces the expression of cyclooxygenase-2 (COX-2), which is a prominent source of the potent antifibrotic prostaglandins^31^. The activation of HGF, and the subsequent induction of COX-2 and prostaglandins synthesis, has been shown to the anti-fibrotic effect^31,32^.

In the present study, LMWH/PLGA-MPs capable of sustained LMWH release were produced. The LMWH-treated group significantly suppressed lung fibrosis compared with the untreated group in BLM-IP mice. IP induced by inhalation of BLM through the airway causes focal inflammatory/fibrotic lesions around bronchioles, which is not clinically compatible with IP in histopathology. In contrast, the continuous subcutaneous infusion of BLM using an osmotic minipump forms inflammatory/fibrotic lesions mainly on pleural side, which is suitable for an IP model. The mechanisms by which LMWH suppressed lung fibrosis were suggested to be suppression of fibrosis formation by HGF and extracellular matrix (ECM)-degrading enzymes. The antifibrotic effect of LMWH has been experimentally confirmed in mouse models of CCl_4_-induced hepatitis^22^ and unilateral ureteral obstruction kidney fibrosis^33,34^.

We conducted animal experiments by using ICR mice, which are easy to administration of PLGA-MPs via the tail vein. A pulmonary fibrosis model can be prepared using this outbred mice by administering bleomycin similarly to inbred C57BL/6 mice^35^. In qPCR analyses using lung tissue, LMWH/PLGA-MP administration significantly suppressed the gene expression of *COL1α1* and *TIMP-1* in the BLM-IP mouse lung. These findings indicate that LMWH suppressed the gene expression of fibrosis-related factors involved in epithelial and endothelial cell injury, fibroblast proliferation, and abnormal repair in lung tissue. In lungs with IP, MMPs are activated during the process of tissue repair and there is a mechanism for ECM degradation^30,36,37^. In the present study, BLM tended to promote *MMP* gene expression in lung tissue, but there was no significant difference compared with normal mice. Possibly because of this effect, LMWH/PLGA-MP administration tended to suppress *MMP-2* gene expression, but there was no significant difference, and *MMP-9* gene expression remained unchanged. A reason why the expression of MMP genes in lung tissue was not significantly promoted by BLM may be that the expression levels were only evaluated in one phase during the time course of IP lung fibrosis. In the future, it may be useful to evaluate the expression at various time phases during the time course of IP lung fibrosis. The expression level of inflammatory cytokine genes tended to decrease in the LMWH/PLGA-MP administration, but there was no significant difference against BLM-alone (Fig. 5G,H,J). A more detailed anti-inflammatory effect of LMWH/PLGA-MP may be evaluated by early phase of inflammation.

The dose of a drug can be reduced and its side effects can be alleviated using PLGA MPs^38^. In BLM-IP mice, gene expression of inflammatory cytokines in lung tissue was enhanced. However, LMWH/PLGA-MP administration did not further enhance the gene expression of inflammatory cytokines. These findings suggest that LMWH/PLGA-MPs did not cause inflammation in the lungs of the BLM-IP mice evaluated in the present study.

## Conclusions

When administered alone, almost all LMWH exhibits anticoagulant activity. In the present study, LMWH showed anti-inflammatory activity, through the effects of LMWH/PLGA-MPs on cells at sites of inflammation. The LMWH/PLGA-MP administration did not further enhance the gene expression of inflammatory cytokines. Combinations of LMWH/PLGA-MPs and anti-inflammatory drugs or mesenchymal stem cells with anti-inflammatory activity may be more effective as anti-inflammatory treatment systems.

## Data Availability

The data that support the findings of this study are available from the corresponding author upon reasonable request.
